# A Study in Materia Medica

**Published:** 1890-07

**Authors:** J. T. Kent

**Affiliations:** Philadelphia, Pa.


### A STUDY IN MATERIA MEDICA.

J. T. Kent, M. D., Philadelphia, Pa.

There is a physician in this city, or at least he has a sign on his door, going about day and night seemingly not in his right mind, or if he be perfectly sane, what he does and says might be attributed to buffoonery (Stram.) with desire to calumniate (Ipec.), but if a very generous view be taken of the matter, he is not responsible for his words and conduct. He bellows on the street (Bell., Canth.), and assumes an air of importance (Hyos., Stram.). Some of his friends have observed great anxiety with sweat (Ars., Graph.). There is great awkwardness about his movements and he drops things (Apis). He is advanced in years prematurely (Bar.-c., Ant.-c.); he is said to be astute in his madness (Anac.), and is much worse in his mental aberrations when alone (Elaps., Phos., or Stram.) with no one to talk to. He is given to alternations of humor (Ignatia), *i. e.*, irritability with cowardice (Ran.-bulb.). He is very jealous (Hyos.) and seems to have an aversion to his own business (Sepia or Kali.-c.) because he attends so diligently to that of others. He has not manifested any desire to destroy his own clothing, but often rips his neighbor's coat up the back (Verat.). In all his ravings he is tearless, yet he is anxious from a slight noise (Caust., Silic., or Aurum), and he seems to dread a storm (Nat.-c., Phos.). He has at times shown great apprehensiveness (Hyos.) with an active cerebral hyperæmia (Glon.). He sees faces from every corner (Phos.), and was known to make rapid movements in the street at the sight of a hand-organ (Phos.-ac.), so great is his aversion to music. Sometimes he thinks he sees cats (Puls., Stram.) and is said to be childish in his behavior (Crocus). Again he imagines he sees far into the future (Acon., Phos.-ac.), and his comprehension is decidedly difficult (Lyc.) especially of what he hears (Cham., Nat.-c.). He frequently manifests a lack of self-confidence (Bar.-c., Kali.-c.), because he knows that there are people living who know the real cause of his insanity (Phos.). Occasionally his conscience troubles him (Ars., Cocc.), and a small boy frightened him the other day by saying "rats!" (Calc.). He often looks back as if pursued by enemies (Dros., Lach.). He went home and looked in the looking-glass and thought he saw a goose (Hyos.). At times he is of a slanderous turn of mind (Nux) and lacking in moral feeling (Anac.). His pride is wonderful (Plat.). He often walks in his sleep (Phos.) and starts at a slight noise (Borax) and has a dread of thieves (Ars., Lach.). Perhaps a nosode would cure him if the product of his disease could be run through Dr. Swan's potentizer. The remedy that causes the totality of symptoms does not appear, even after long study. Even "Christian Science" has failed to make a man of him. It has recently been reported that he has resorted to stimulants, and still he fails. Is there no saving a man who will not save himself? Echo answers, "no saving!"—*Journal of Homœopathics, May No., page* 13.

